# Fractal-like geometry as an evolutionary response to predation?

**DOI:** 10.1126/sciadv.adh0480

**Published:** 2023-07-26

**Authors:** Robert Lemanis, Igor Zlotnikov

**Affiliations:** BCUBE - Center for Molecular Bioengineering, Technische Universität Dresden, Dresden 01307, Germany.

## Abstract

Fractal-like, intricate morphologies are known to exhibit beneficial mechanical behavior in various engineering and technological domains. The evolution of fractal-like, internal walls of ammonoid cephalopod shells represent one of the most clear evolutionary trends toward complexity in biology, but the driver behind their iterative evolution has remained unanswered since the first hypotheses introduced in the early 1800s. We show a clear correlation between the fractal-like morphology and structural stability. Using linear and nonlinear computational mechanical simulations, we demonstrate that the increase in the complexity of septal geometry leads to a substantial increase in the mechanical stability of the entire shell. We hypothesize that the observed tendency is a driving force toward the evolution of the higher complexity of ammonoid septa, providing the animals with superior structural support and protection against predation. Resolving the adaptational value of this unique trait is vital to fully comprehend the intricate evolutionary trends between morphology, ecological shifts, and mass extinctions through Earth’s history.

## Teaser

Fractal-like shapes of ammonoid septa provide the animals with enhanced structural stability and protection against predation.

## INTRODUCTION

The recognition of fractal-like systems in biology has a long and varied history (*1*), from the shape of taxonomic patterns (*2*) to microbial growth and metabolic scaling (*3*, *4*). The exploitation of fractal structures has a clear benefit for respiratory organs by maximizing the surface area of the functional tissue and minimizing volume (*5*). In engineering, fractal-like morphologies can increase a structures resistance to compressive loads (*6*), improve flexural strength (*7*) and increase energy absorption (*8*, *9*). However, can fractal-like morphologies render similar solutions to living organisms, providing them with structural support, or in other words, can the evolution of fractal-like geometries be driven by functional adaptation toward superior mechanical performance? The iterative evolution of the fractal-like morphology of the inner architecture of ammonoid cephalopods presents an ideal system to answer this question.

The shells of ammonoid cephalopods occur throughout approximately 351 million years of Earth’s history (*10*). Over this period of time, ammonoids would have seen both small-scale and large-scale changes in environments and ecology, including the formation and eventual break-up of the supercontinent Pangea (*11*) and four of the so called Big Five mass extinctions through the Phanerozoic (*12*). Their global distribution, high abundance, and long persistence through time make ammonoids excellent subjects to study paleoecology and evolutionary dynamics. However, to fully understand the information locked away in these shells, we first have to have an understanding of basic aspects of their biology. Unfortunately, some of these fundamental questions in ammonoid paleobiology remain unanswered. For example, the morphology of the arm crown remains largely unknown and long-standing hypotheses about the connection between shell shape, hydrodynamics, and ecology have only recently begun to be quantified and tested (*13*–*18*) since earlier work in the late 1900s (*19*–*24*). Nevertheless, what might be the longest-lasting question with regard to ammonoid biology, the question of the function of the fractal-like septa, remains open.

**Fig. 1.**
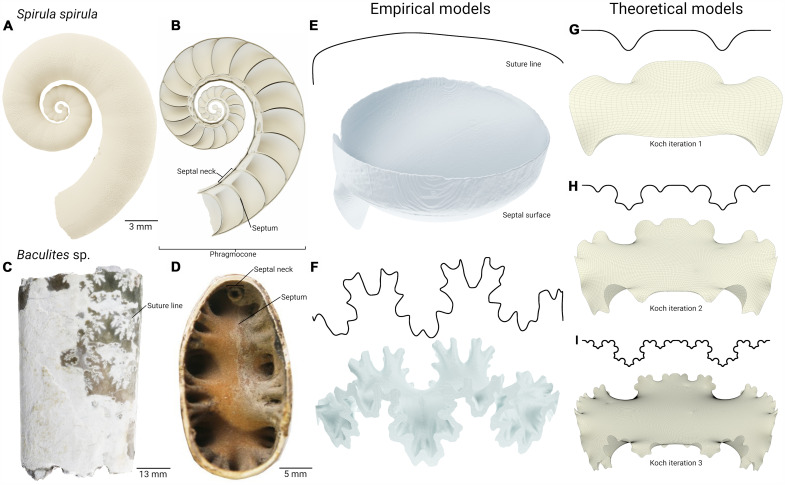
Overview of the shell structure of cephalopods and septal morphology. (**A**) Three-dimensional (3D) rendering of the shell of *Spirula spirula*. (**B**) Cross section of the same *Spirula* shell showing the internal morphology of the phragmocone, including the septa and the septal necks that surround the siphuncle. (**C**) A surface scan of a *Baculites*, more complete than the specimen used in this study, from the Pierre Shale of South Dakota from the Paleontological Research Institution, New York. (**D**) Top-down view of the septa of the *Baculites* specimen used in this study detailing the equivalent features shown in *Spirula* (B). (**E** and **F**) Suture lines and septal surface renderings created from computed tomographic data of the two specimens used in this study. Suture lines are created from tracing the edges of the surface renderings and projecting them onto a 2D plane. (**G** to **I**) Simulated suture lines and septa created from Koch curves of increasing fractal order. Septa are formed as minimum curvature surfaces using the suture lines as a constraint. Koch septa are simplified in that they lack septal necks and the siphuncular foramen. The models used in this study consist of the shell tube with three septa and a mechanical stress directed on the middle septum.

The septa are mineralized walls within the shell tube of cephalopods (Fig. 1, A to D) and some gastropods, creating their internal chambers, although, in gastropods, they appear to be nonfunctional (*25*). The cephalopod septum is penetrated by an organic strand that permits the diffusion of fluids into and out of the chambers, allowing the cephalopod shell to achieve its primary function as a buoyancy apparatus. Tracing the attachment of the septum and the shell wall creates a suture line (Fig. 1, E and F). The complexification of the ammonitic suture line can be illustrated by contrasting an early septum of *Baculites* (Fig. 1F) to the much simpler septum of the modern *Spirula* (Fig. 1E). The driver(s) of this complexification has been the subject of intense speculation.

The most recent review of ammonoid paleobiology lists 12 hypotheses extracted from over a century of literature concerning the potential functions of the ammonitic septum (*26*). Not all of these hypotheses are equally likely. For example, the suggestion by Pia (*27*) that the complexity of the septum is driven by the need to rapidly construct a rigid shell to bound the gas within the chamber lest it push the animal out of the shell, is borne by the idea of high pressure within said chamber, an idea that is now known to be false (*28*, *29*). Other ideas, such as the Cartesian diver model (*30*), while provocative, have never been particularly convincing (*31*). Mechanical hypotheses have historically related septal complexity to buttressing, helping to prevent the shell from imploding because of water pressure (*32*, *33*). However, this interpretation is increasingly doubtful because of both mechanical considerations of the septa under hydrostatic pressure (*34*, *35*) and a lack of any consistent correlation between complexity and habitat depth (*36*–*38*). Hydrostatic pressure was the dominant suggested evolutionary driver in much of the literature and largely the only “mechanical,” as opposed to “physiological,” hypothesis discussed. However, in some cases where hydrostatic pressure was clearly not a concern, for example, in the shallow water habitat of fractal-like *Placenticeras*, predation would be invoked (*39*). Predation was an ancillary mechanical hypothesis that was often only mentioned alongside a hydrostatic pressure (*33*, *40*). Predation was not even one of the previously mentioned 12 hypotheses of septal function. Using comparative mechanics, we make the argument here for predation as the primary mechanical hypotheses for the complexification of the ammonitic suture line, and moreover, a prime example of how fractal-like geometry can be exploited for functional reasons by biological systems.

One of the first applications of finite element analysis in paleontology was the investigation of cephalopod shell strength (*41*, *42*). In the intervening decades, a number of studies have provided no shortage of stress and strain values across a diverse range of shell and septal morphologies (*34*, *35*, *40*, *43*) from which very few strong conclusions could be drawn. Even in cases where systematic increase in complexity is correlated with changes in strength, these effects tended to be small. For example, increasing septal complexity was shown to correlate to a minor decrease of tensile stresses, equivalent only to a minor increase in depth tolerance of around 30 meters (*35*). We suggest the focus on relative strength, all derived from linear static simulations, might be a red herring. While septal frilling might be nonfunctional, a kind of fabricational noise, we think there are good reasons, which are examined later in the discussion, to believe the trend toward higher complexity is adaptational. Previous results suggest other factors influencing the functionality of these structures, demanding the search for parameters beyond tensile strength. Buckling is an important phenomenon, as it can cause structural failure well before the tensile or compressive strength of a material is reached. The critical load in buckling analysis represents the transition from a stable state to an unstable one. In this case, a stable structure is able to respond to the compressive load elastically once the load is removed, returning to its original shape. Once the load goes beyond the critical point, the structure will continue to deform beyond recovery and will no longer be able to function. The force needed to induce this instability is the main parameter extracted from our buckling analyses. Several papers have discussed buckling in cephalopod shells, although largely only from compression via hydrostatic pressure (*44*–*46*). Here, we extend this discussion to include computational simulations of septal buckling under point loads. Combining the partially neglected topic of predation with a suite of mechanical simulations that have yet to be applied to septa, we uncover a clear relationship between septal complexity and resistance against buckling.

## RESULTS

### Model creation and loading

A combination of theoretical and computed tomography (CT)–derived, empirical models (Fig. 1, E to I) of septal morphologies were combined with linear and nonlinear finite element simulations of structural buckling to test shell resistance against a simulated predator bite. The use of theoretical models allowed us to follow the performance trends of a systematically increasing morphological complexity that would not be possible in actual shells. Prior work differentiated primary and higher-order complexity, where primary septal complexity is the folding that characterizes the primary suture line (*35*, *47*). Higher-order complexity was modeled through an adaptation of the well-known Koch fractal curves as the boundary conditions to calculate minimum curvature surfaces that form the basis for the modeled septa (Fig. 1, G to I). While valuable, models such as these necessarily simplify true morphology and morphological relationships. To supplement these models, CT data are used to create finite element models of two “end-member” morphologies: the simple, domic septa of *Spirula spirula*, and the ammonitic septa of *Baculites* sp. (Fig. 1, E and F, respectively).

These models were subjected to three sets of simulations: linear prebuckling, linear static, and nonlinear postbuckling. Linear prebuckling analysis calculates the relative displacement of the structure under the applied load and provides the force needed to destabilize the structure. Linear static simulations were performed on the original, undeformed geometries to provide maximum principal stress and strain energy values for these models. These results were then compared to the nonlinear postbuckling simulations that provide maximum principal stress and strain energy of the buckled geometries. Deformed models for the postbuckling analyses were constructed using displacement data from the linear buckling simulations. The relative displacement from the different buckling modes was then extracted and applied as perturbation inputs for the postbuckling simulations to create models whose initial geometry is slightly deformed compared to the original models by the previously applied buckling load in the prebuckling analysis. Ultimately, the comparison between the linear static and the nonlinear postbuckling simulations demonstrates how slight geometric imperfections in the structure affect the stress and strain under the same loading conditions.

### Linear buckling in theoretical models

**Fig. 2.**
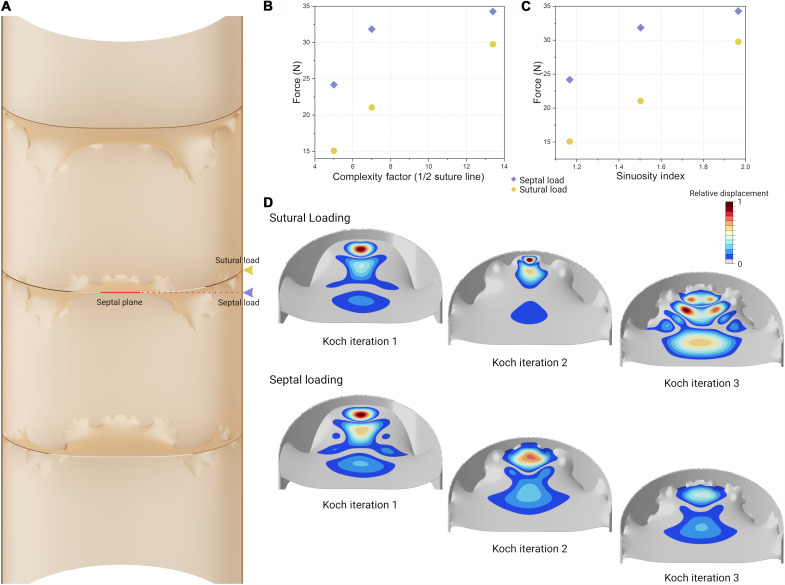
Increasing septal complexity increases the critical load to induce instability. (**A**) The loading scheme used to test the theoretical Koch models against simulated predator bites on the outer shell wall. The theoretical septa are placed inside a cylindrical shell model that has been bisected in this view to show the placement of the septa, just as in Fig. 1B. Sutural loads are directed along the direct attachment site of the septa to the shell wall. Septal loads are directed along the projected plane of the middle of the septum. (**B** and **C**) Graphs of complexity against the force needed to induce instability, calculated from the first buckling mode of the linear buckling simulations. Complexity factor is calculated by assigning first-order folds a value of 1 and higher order folds a value of 0.1 and adding them together. The sinuosity index is the ratio between the inner circumference of the shell tube and the total path length of the suture line. (**D**) Relative displacement contour maps are produced from the linear buckling simulation results. Here, the loaded septa are viewed cut out of the larger model (A) to show the region of interest. The part of the septum adjacent to the constraint has also been cropped to improve the visibility of the relevant area and because this region has no displacement. Maximum relative displacement decreases with increasing complexity under septal loading. Under sutural loading, displacement migrates toward the center of the septum.

Three Koch model iterations with increasing suture line complexities (Figs. 1, G to I) were loaded at two locations along the suture: the septal load that is incident on the shell wall within the projected plane of the center of the septum and the sutural load that is incident on the point of direct contact between the septum and the shell wall (Fig. 2A). Complexity is not a rigorously defined concept for suture lines; for this reason, two different complexity metrics are taken to compare the relative performance between models. For the first buckling mode, complexity factor (Fig. 2B) and sinuosity index (Fig. 2C) all show the same trend: As complexity increases, the force needed to destabilize the structure increases. Comparing the simplest against the most complex theoretical septal morphology, the needed force to destabilize the structure along the septal plane increases by 42% while the necessary force along the suture increases by 97% (table S1). Correlation coefficients between force and complexity for these models range from 0.83 to 0.99. Furthermore, increasing complexity shifts the zone of peak displacement away from the edge of the septum and toward the center when loading along the suture line (Fig. 2D). Under septal loading, the relative displacement does not show much of a change in location but a decrease in magnitude with increasing complexity (Fig. 2D).

### Pre- and postbuckling of theoretical models

Linear static simulations on the Koch models show how the geometry of the septum near the load application point affects the deformation and stress distribution (Fig. 2D). The broad, low curvature of the first iteration saddle (the folds that point toward the aperture as opposed to lobes that point toward the shell apex) transmits the highest stresses to the flanks of the neighboring saddles. As this flat region decreases in size in the second iteration model, the stress becomes concentrated in this zone. Here, high tensile stresses are formed as the load on the opposite side of the septum creates a strong bending moment about the attachment site. The additional third-order folds added to this region in the third iteration model break up this linear zone of high stress and direct some of the stresses onto the septum face. The same patterns are broadly recreated after applying the buckling deformation in the nonlinear postbuckling models, with one difference being in the first iteration model where sutural loading creates a zone of high stress near the loading site. Furthermore, the iteration 2 and 3 models show bands of stress fanning outward from the loading site toward the center of the septum that arc between opposing saddles. The main difference, however, between the static and postbuckling simulations is the magnitude of stress. Introducing 2% deformation from the buckling simulations increases the calculated peak tensile stresses up to 66-fold in the Koch models, compared across all loading conditions (tables S2 and S3). This outcome emphasizes the importance of performing nonlinear postbuckling simulations, but most importantly, it demonstrates the critical importance of a buckling event that the structure would need to avoid to prevent failure.

### Simulated behavior of empirical models

Expanding the comparisons to the models of *Spirula* and *Baculites*, the trends are similar to those in the theoretical models. The shell of *Baculites* show substantially more stability compared to *Spirula* (table S1). Similar to the Koch models, the magnitude of the eigenvalues, as well as corresponding forces, increases as the load moves from the adoral/adapical-most point of the suture line toward the septal plane; note that the loading locations are not equivalent between the Koch models and those of *Spirula* and *Baculites* because of the morphology of the septum. Comparing buckling of the attachment zone between the two cephalopods, *Baculites* requires an average of 38% more force (a difference of 1334 N) to destabilize the structure compared to the simpler septa of *Spirula*. This is true despite the fact that the septum of *Baculites* is less than half the thickness of *Spirula*: 0.5 mm (±0.8) versus 1.2 mm (±0.06). The more elliptical whorl shape of *Baculites* will also have an effect on the buckling load compared to the more circular whorl shape of *Spirula*. However, comparing the first eigenvalues from a load directed on the side of the *Baculites* shell wall against a load on the bottom of the shell wall (opposite the siphuncle), the difference is only around 4%.

**Fig. 3.**
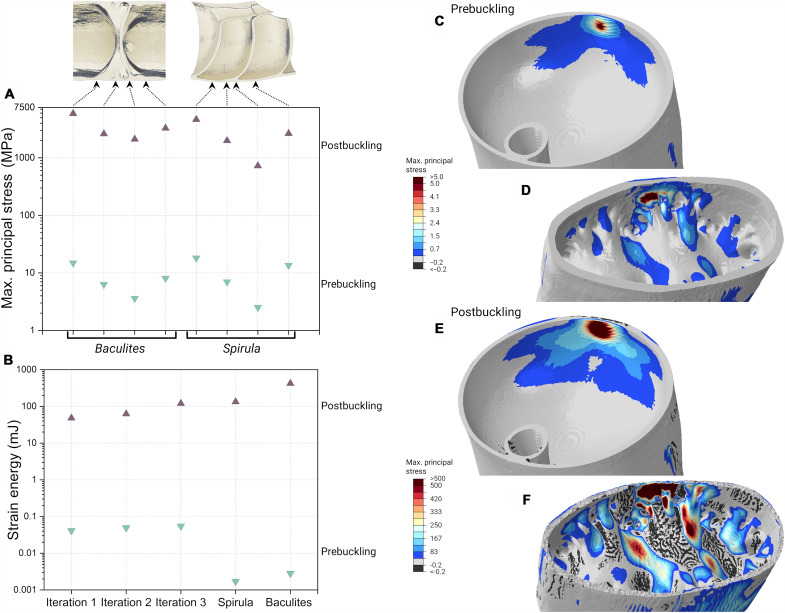
Comparing performance between *Spirula* and *Baculites*. (**A**) Maximum principal stress across the four loading locations in both models. Prebuckling analysis is from the undeformed models, while postbuckling analysis is performed by applying some of the deformation from buckling analysis to the original geometry to test the effects of deformation on the performance of the structure. Adding this deformation greatly increases the stress developed in the structure under equivalent loading conditions. The location of the loads is shown in the coronal section of *Baculites* and in the median section of *Spirula*. (**B**) Total strain energy values from all models used in this study. Strain energy increases with increasing complexity. The difference in prebuckling values between theoretical and empirical models is likely due to empirical models being much thicker than the theoretical models. (**C** to **F**) Stress contour maps of *Spirula* and *Baculites*. While *Spirula* shows a subcircular stress pattern from point loading, *Baculites* shows a more complex pattern; stress develops both around the point of load application and is transmitted to areas of high curvature in the adjacent folds and near the center of the septum.

The models of *Baculites* and *Spirula* show even greater increases in the postbuckling analysis, with the highest stresses increasing around 580-fold (Fig. 3A and tables S2 and S3). Here, the data again emphasize that the septal geometry has little influence on the stresses developed in the shell; the range of stress values for the prebuckling simulations shows a similar range between *Baculites* and *Spirula*. Increasing complexity in both empirical and theoretical models also increases the strain energy stored in the structure (Fig. 3B). Postbuckling strain energies uniformly increase for all models but seem to increase at a greater rate for more complex models. Stress contours show a greater sensitivity to geometry in *Baculites* compared to *Spirula*. Whereas stress contours are generally circular regardless of load location in *Spirula* (Fig. 3, C and E), in *Baculites*, they tend to follow the shape of the arced folds or appear on nearby high-curvature regions (Fig. 3, D and F). Stress and displacement also get some component transferred to the central area of the septa, something not seen in *Spirula*.

Both theoretical and empirical models show that increasing septal complexity increases energy absorption and the force needed to induce instability and that small deformations from buckling-induced shape changes can fatally increase the stress in the structure. This creates a strong incentive to prevent this deformation in the first place by increasing the force needed to buckle the structure. Septa with low geometric complexity will buckle at notably lower forces than more complex septa, both in the case of the theoretical and empirical models.

## DISCUSSION

Finite element analyses on theoretical and empirical cephalopod shell models reveal a clear relationship between septal morphology and buckling behavior. Increasing the complexity of the septum increases stiffness and elastic energy absorption, and increases the force necessary to destabilize the septum (Figs. 2 and 3). The clarity of this relationship underscores the proposed hypothetical function of ammonite-like septa as responses to predation.

Consider the phragmocone, it is a vital structure for externally shelled cephalopods, as it allows the animal to overcome the weight of its own body and ascend off the ocean floor and into the water column. The primary function of septa, therefore, is to create a rigid container that can be filled with gas such that the gas volume is not compressed by water pressure. Why then did ammonoids uniquely evolve such highly folded septal structures not seen in their sister clade Nautiloidea? There are several reasons to believe that there are functional explanations behind the evolution of this trait rather than just, for example, fabrication or drift.

First, there is no reason to believe that the production of a shell, even the highly heteromorphic shells of Ancyloceratina (*48*), requires a complex septum. Nautiloids and gastropods can manufacture similar shells to ammonoids without complex septa or without septa at all in the latter case. Theoretical models for shell formation also do not require any term for the septa (*49*–*52*) to model realistic shell growth. Second, the evolutionary trend toward increasing sutural complexity is one of the most frequent trends observable in ammonoids (*53*–*55*). This observation includes not only the increase of maximum observed complexity but also an increase of minimum observed complexity (*56*), which is what would be expected of a driven, directional trend (*57*). This was also not a singular event; the evolution of complexly folded septa occurs multiple times in different periods and within different clades. More complex forms seem to be selected against across certain extinction events (*58*, *59*), such as the Frasnian-Famennian and Permian-Triassic (*56*), and illustrated by the evolution of the more simple *Psiloceras* from more complex Phylloceratoids across the Triassic-Jurassic boundary (*60*). The recurring trend toward complexity coupled with the uniqueness of the ammonitic suture line implies functionality.

Suture line morphology also shows some sensitivity to environment, such as transgression-regression cycles, although this can be a complex signal (*22*, *61*, *62*). Regardless, if the ammonitic-type septal morphology is functional, it renders the structures multifunctional, as their primary purpose is, again, the creation of a rigid body for buoyancy. Other buoyancy system–related functions have been proposed for higher-order complexity, such as improved fluid retention via water tension (*63*) that might contribute to the complex evolutionary dynamics of the group. The primacy of buoyancy in considerations of septal functionality underlies the importance of the phragmocone for the animal.

The shell is formed by the mantle, which, in cephalopods, is situated entirely in the body chamber. Therefore, if the shell is damaged, the only portion of the shell that can be repaired are the portions in direct proximity to the mantle, i.e., the body chamber, the most recently formed septum, and the small portion of the phragmocone immediately in front of the shell aperture (*64*–*66*). It stands to reason then that ensuring the structural integrity of the phragmocone is of extreme importance for the continued survival of the animal because once the shell wall breaks, the shell will flood and will likely result in death.

Furthermore, while reconstructing food webs in the fossil record is a complicated task, direct evidence through stomach contents show that ammonoids were preyed upon by both invertebrates and vertebrates. Common predators of ammonoids were other cephalopods, as evidenced by small aptychi in the digestive tract of other larger ammonites (*67*–*69*) and some coleoids (*70*). The durophagus pycnodontid fish *Gyrodus* from the Late Jurassic was found with aptychi in its stomach (*71*). Ammonite remains are also found in marine reptiles such as ichthyosaurs (*72*) and metriorhynchids (*73*). In addition, window-like breakages in some shells have been interpreted as damage from stomatopod crustaceans (*74*). The point loads generated by crustaceans, the sharp beaks of coleoids, or the teeth of fish and marine reptiles (*75*) could represent an important driver for greater shell resilience.

Recently, the potential relationship between durophagus predators and septal morphology was investigated using a series of three-dimensional (3D) printed models loading under compression (*76*). Although, as pointed out above, it was concluded that different septal morphologies do not have a large impact on compressional strength, their results revealed an interesting difference in failure patterns. The simple, nautiloid-type morphologies would fail through the entire structure, breaking the shell wall. Conversely, more complex septa tend to localize failure in the septum exclusively, leaving the shell wall intact (*76*). In the context of the inability of the animal to repair the phragmocone, this behavior can be interpreted as beneficial because it can change potentially fatal structural failure into a nonfatal form of failure. Although this work does not test this specifically, we demonstrate a correlation between more complex morphologies and the transmittance of displacement, as well as some stress, toward the center of the septum (Figs. 2D and 3, C to F). The results presented here also do not show any specific relationship between complexity and relative strength (table S1), but the data reveal a clear relationship between structural stability and septal complexity.

Although septal “complexity” has no strict definition, all morphometric definitions of complexity tested here show the same trend: The greater the complexity, the stiffer the structure and the greater the force needed to destabilize the septum (Fig. 2, B and C). Even small amounts of deformation after septal buckling can increase the stress in the shell by orders of magnitude (Fig. 3). This massive increase shows a clear vulnerability to deformation in these brittle shells, the failure of which would be lethal for the animals. Hence, unlike maximal stresses, resistance to predation-induced buckling presents a notable driving force that might explain the iterative evolution of complexity and the emergence of fractal-like septal morphologies. This relationship can be further investigated in the future with high-resolution stratigraphic work and high-resolution ecological reconstructions looking at the predator appearances and diversity against ammonite morphological changes.

Last, the relationship between septal complexity and buckling could not be clarified in classical linear elastic analyses that are common in paleontology, demonstrating the importance of including both nonlinear simulations and other types of computational mechanical tests to further reveal potential relationships between morphology and function that has recently been argued for (*77*). If septal complexity is related to escalation (*78*), then they demonstrate how biological systems can exploit fractal morphology not only for physiological reasons (*5*) but also in terms of solid mechanics, linking biology and engineering (*6*–*9*) and providing inspiration for resilient structural design.

## MATERIALS AND METHODS

### Theoretical models

The theoretical models used here are the same as those used in previous research (*35*). Models components (septa, cylinders, and hemispherical caps) were created and assembled in the program Rhino3D. The initial curve used to construct the theoretical suture line was created from smoothed Koch curves of up to three iterations. This created a single “lobe” that was then duplicated four times to create the entire suture line. The complete suture line was imported into Inkscape where they were converted into vector image files. The suture lines are imported into Rhino3D and wrapped around cylinders of equal radius. The two ends of the curve are joined together to form a single, closed curve. Septal surfaces were constructed using an algorithm, implemented in grasshopper, which approximates a minimum curvature surface. These surfaces were then used to construct septa of equal thickness (0.04 mm) that were combined with the modeled cylinders and caps via Boolean unions. The resulting complete surface was exported into Avizo to refine the mesh, correct defects, and create a tetrahedral mesh.

### CT scanning

The shell of *S. spirula* was scanned at the Department of Operative and Preventative Dentistry of the Charité, Berlin, with a Bruker SkyScan 1172. The collected data were reconstructed with NRecon, resulting in a tomographic stack with an isotropic voxel size of 4.9 μm (*79*). The shell of *Baculites* sp. (RUB-PAL 11275) was scanned at the GeoForschungsZentrum in Potsdam, with a Phoenix Nanotom S. The shell was reconstructed with an isotropic voxel size of 19.4 μm. Tetrahedral meshes were created from both CT datasets using Avizo. This specimen consists of a single chamber bounded by two septa. The best-preserved septa and the surrounding shell were segmented and duplicated twice to create a larger model and move the finite element constraints farther from the region of interest. The original *Baculites* shell shows additional material on the inner surface, which artificially thickens the specimen. To correct for this, the entire shell was thinned so the average thickness values fell within a previously measured range for *Baculites compressus* of equivalent whorl height (*80*).

### Finite element analysis

All finite element simulations were performed in Abaqus. Three sets of simulations were performed for all models: linear static, linear buckling, and nonlinear postbuckling simulations. Postbuckling simulations were solved using a general Riks solver. All models had an isotropic elastic modulus of 70 GPa with a Poisson’s ratio of 0.3. A pressure load, with a total force of 5 N, was applied to all models over a small, hexagonal cluster of elements to avoid stress singularities that can arise from applying force at a single node. Values for peak displacement and maximum principal stress are taken from the septa and mural zones. The ten highest values are extracted from the models and averaged together. Values from the peak contour immediately around the applied force are excluded to avoid potentially inflated values.

Constraints for the theoretical models consisted of a line of nodal constraints, opposite the applied load, against translation in the direction parallel to the loading direction; additional lateral (with respect to the load) nodal constraints were added to prevent translation in the direction perpendicular to the load (fig. S1). This was done to prevent “rolling” of the model due to slight mismatches between the loading axis and the opposite constraint location. Sensitivity analysis performed on the iteration 2 model showed that the first eigenvalue from the buckling analysis was due to this rolling behavior, although the second eigenvalue showed the anticipated deformation and was equivalent to the first eigenvalue of the model with antirolling constraints (table S4). The same constraints were used for the *Spirula* and *Baculites* models. Because both shells were cut out of the full shell, the planes formed by the cut were additionally constrained against perpendicular motion. The *Spirula* model was scaled up to the same shell volume as the *Baculites* model to eliminate potential size effects between the two models.

All loads are located on the external shell surface. Koch models were all loaded in two homologous locations. The first location is taken by extrapolating a line, parallel to the flat region in the center of the septum in the median section, to the shell wall to create the septal load (Fig. 2A). The sutural load is the point in which the septum physically attaches to the shell wall (Fig. 2A). *Spirula* and *Baculites* are loaded in four locations (Fig 3A). *Spirula* has one load directed on an unsupported section of the shell wall, two loads at the septum-shell wall attachment site (the adoral most point at the tip of the attachment site and the apical base of the attachment site), and the final load on the septal plane axis similar to the Koch models. The complexity of the *Baculites* shell leads to different loading conditions relative to *Spirula*. In the case of *Baculites*, the attachment zone was the main interest. In the coronal section, the loads are along the attachment site: the adoral tip of the attachment site (saddle), the apical tip of the attachment zone (lobe), the point at which the visible septum attaches to the wall, and a point approximately midway between this point and the saddle tip (Fig. 3A).

All models were meshed with quadratic tetrahedral elements. Convergence testing was done on the basis of maximum principal stress values with a tolerance of ±10% to satisfy the convergence criterion; more about the mesh accuracy of the Koch models is given in a previous paper (*35*). Additional sensitivity analysis was done with the postbuckling Riks analysis. The results of the linear buckling analyses were used as perturbation inputs for the Riks calculations. The first three buckling modes were used to introduce the deformation into the Riks model. Two different models, created from the iteration 1 model, were created using different degrees of deformation inherited from the first three buckling modes: 20%/15%/10% and 2%/1.5%/1%, respectively. Both models show large increases in stress values compared to the linear static analyses. Peak maximum principal stress values increased by 1258% in the 2%/1.5%/1% and 1342% in the 20%/15%/10% model. Ultimately, results from the 2%/1.5%/1% model were used for this work.

Linear buckling simulations were solved using the Lanczos method with a minimum eigenvalue of 1 × 10^−6^ to eliminate negative eigenvalues. Riks simulations were limited to 50 increments for the Koch models and 30 increments for the *Spirula* and *Baculites* models because of long solution times. The eigenvalue problem solved in the linear solver is represented by

$$\left(K_{0}^{NM}+\lambda_{i}K_{\text{Δ}}^{NM}\right)\nu_{i}^{M}=0$$

where $K_{0}^{NM}$is the initial stiffness matrix and $K_{\text{Δ}}^{NM}$ is the stiffness matrix generated from applying an incremental loading pattern. λ and ν are the eigenvalue and eigenvector, respectively. *M* and *N* are the degrees of freedom, and *i* is the *i*th buckling mode (*81*). The eigenvalue is the load multiplier, while the eigenvector is the buckling mode. In general, the lowest (first) buckling mode is the primary interest. Higher buckling modes play a role if buckling in the first mode is prevented. Most comparisons between buckling values used here are taken from the first buckling mode.

### Suture complexity

The complexity factor (*82*) is calculated using the half-suture line, while the sinuosity index is calculated from the entire suture line path length against the inner circumference of the shell.
