# Supplementary material for: Fractal-like geometry as an evolutionary response to predation?
Source: Sci Adv. 2023 Jul 26;9(30):eadh0480. doi: 10.1126/sciadv.adh0480 (PMC10371019; doi:10.1126/sciadv.adh0480)
Supplement: Supplementary file 1 — Fig. S1 Tables S1 to S4 [file sciadv.adh0480_sm.pdf]

Science Advances
AAAS

# Supplementary Materials for

## Fractal-like geometry as an evolutionary response to predation?

Robert Lemanis *et al.*

Corresponding author: Robert Lemanis, robert_evan.lemanis@tu-dresden.de

*Sci. Adv.* **9**, eadh0480 (2023)
DOI: 10.1126/sciadv.adh0480

**This PDF file includes:**

Fig. S1
Tables S1 to S4

**Figure S1.**

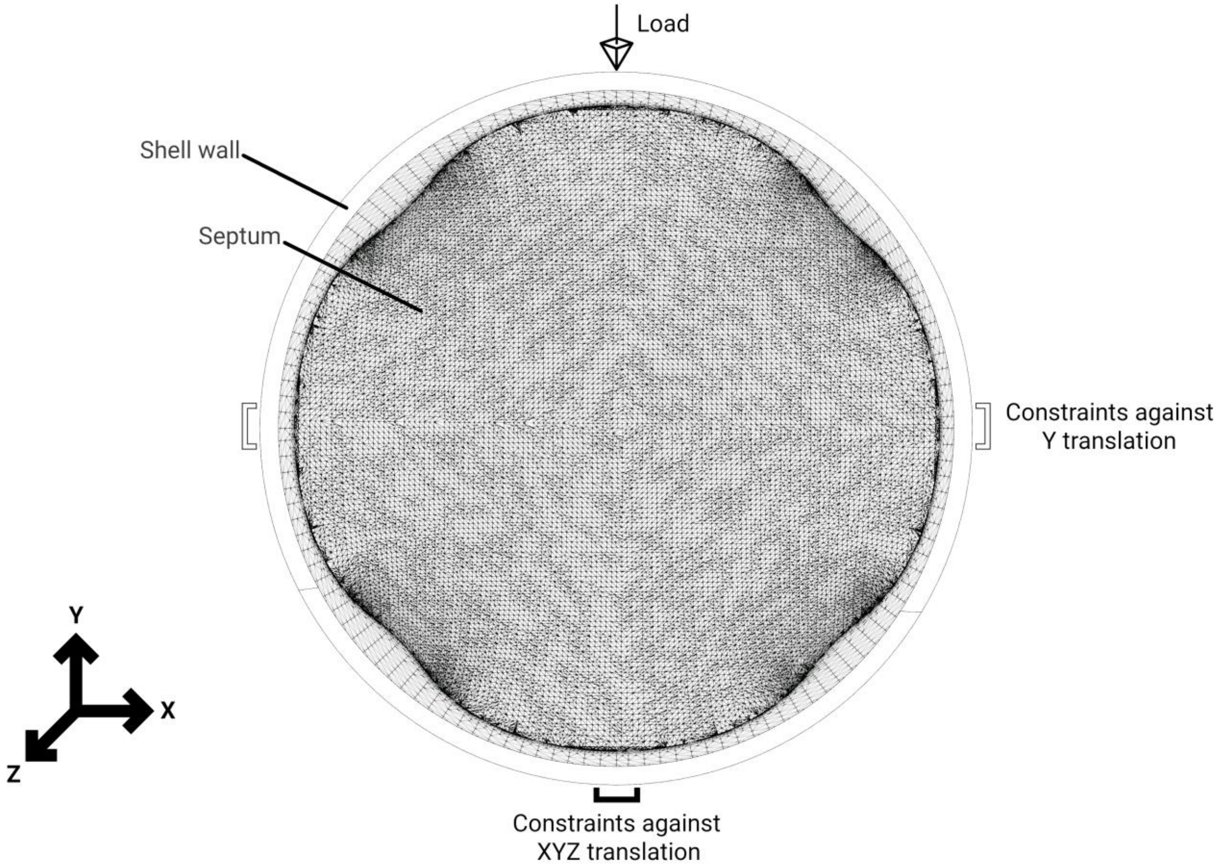


**Representative boundary conditions for all models in this study.** A small area at specific locations in each shell model was chosen as the loading site, representing the contact area between a potential predator (tooth, beak, etc.) and the shell. Opposite the applied load are the main constraints that prevent translating in all three directions. Lateral constraints are imposed to prevent the model from rolling relative to the bottom constraints. The area of all constraints extends the entire length of the model.

**Table S1.**

| Model | 1st Eigenvalue | 2nd Eigenvalue | 3rd Eigenvalue | 4th Eigenvalue |
|---|---|---|---|---|
| ***Koch Models*** | | | | |
| Septal Load 1st Iteration | 4.8362 | 5.2554 | 7.2525 | 7.3643 |
| Septal Load 2nd Iteration | 6.3680 | 7.0048 | 9.2400 | 9.6430 |
| Septal Load 3rd Iteration | 6.8543 | 7.3346 | 11.2690 | 11.5140 |
| Suture Load 1st Iteration | 3.0173 | 3.2990 | 5.0131 | 5.2184 |
| Suture Load 2nd Iteration | 4.2103 | 4.7135 | 5.2666 | 5.4708 |
| Suture Load 3rd Iteration | 5.9527 | 6.0054 | 6.3183 | 6.3752 |
| ***Spirula*** | | | | |
| Interchamber | 389.65 | 981.86 | 1068.8 | 1188.8 |
| Attach Tip | 615.25 | 961.51 | 1114.1 | 1616.4 |
| Attach Base | 793.26 | 1054 | 1181.1 | 1493.2 |
| Septal Plane | 277.93 | 698.43 | 812.95 | 982.43 |
| ***Baculites*** | | | | |
| Saddle Tip | 672.38 | 1476.9 | 1673.2 | 2256.2 |
| Mid Saddle | 1202.9 | 1800.7 | 1916 | 3300.7 |
| Septal Attachment | 1167.2 | 1741 | 1877 | 3226.2 |
| Lobe | 841.61 | 1607.5 | 1831.8 | 3045 |

**Results of the linear buckling simulations**. Eigenvalues are the critical load multipliers from the buckling simulations. They can be multiplied by the applied load (5 N) to convert them to force.

## Table S2.

| Model | Location | Parameter | Average | Standard Deviation |
|---|---|---|---|---|
| **Koch Iteration 1** | | | | |
| | Septal Plane | Total Strain (mJ) | 0.103407 | |
| | | Displacement (mm) | 0.0540224 | 5.13907E-07 |
| | | Max. Principal Stress (MPa) | 203.8168 | 5.0004095 |
| | Sutural Load | Total Strain (mJ) | 0.041453 | |
| | | Displacement (mm) | 0.06235355 | 6.98928E-07 |
| | | Max. Principal Stress (MPa) | 239.3083 | 7.573706742 |
| **Koch Iteration 2** | | | | |
| | Septal Plane | Total Strain (mJ) | 0.137175 | |
| | | Displacement (mm) | 0.03884138 | 2.45186E-06 |
| | | Max. Principal Stress (MPa) | 321.7513 | 7.957287579 |
| | Sutural Load | Total Strain (mJ) | 0.0494756 | |
| | | Displacement (mm) | 0.04974734 | 2.67514E-06 |
| | | Max. Principal Stress (MPa) | 222.1004 | 2.498082913 |
| **Koch Iteration 3** | | | | |
| | Septal Plane | Total Strain (mJ) | 0.129228 | |
| | | Displacement (mm) | 0.03241272 | 1.81207E-06 |
| | | Max. Principal Stress (MPa) | 223.2511 | 1.837787879 |
| | Sutural Load | Total Strain (mJ) | 0.0545532 | |
| | | Displacement (mm) | 0.04498648 | 1.89304E-06 |
| | | Max. Principal Stress (MPa) | 355.9833 | 11.47315394 |
| ***Spirula*** | | | | |
| | Inter-Chamber | Total Strain (mJ) | 0.00431862 | |
| | | Displacement (mm) | 0.001624834 | 6.7892E-06 |
| | | Max. Principal Stress (MPa) | 18.12545 | 0.138209408 |
| | Septal Attachment | Total Strain (mJ) | 0.00171385 | |
| | | Displacement (mm) | 0.000531426 | 8.26642E-07 |
| | | Max. Principal Stress (MPa) | 6.991661 | 0.166277788 |
| | Septal Attachment Base | Total Strain (mJ) | 0.0011238 | |
| | | Displacement (mm) | 0.000287014 | 1.36486E-06 |
| | | Max. Principal Stress (MPa) | 2.523017 | 0.11700271 |
| | Septal Plane | Total Strain (mJ) | 0.00387368 | |
| | | Displacement (mm) | 0.0013536 | 2.36461E-06 |
| | | Max. Principal Stress (MPa) | 13.5398 | 0.083730174 |
| ***Baculites*** | | | | |
| | Lobe | Total Strain (mJ) | 0.00220088 | |
| | | Displacement (mm) | 0.000796731 | 3.23772E-06 |
| | | Max. Principal Stress (MPa) | 8.063818 | 0.129610997 |
| | Septal Attachment | Total Strain (mJ) | 0.00130806 | |
| | | Displacement (mm) | 0.000399581 | 1.42744E-06 |
| | | Max. Principal Stress (MPa) | 3.604925 | 0.018218075 |
| | Mid Saddle | Total Strain (mJ) | 0.00128011 | |
| | | Displacement (mm) | 0.000422483 | 2.19559E-06 |
| | | Max. Principal Stress (MPa) | 6.335535 | 0.168474307 |
| | Saddle Tip | Total Strain (mJ) | 0.00282073 | |
| | | Displacement (mm) | 0.001030853 | 1.45207E-06 |
| | | Max. Principal Stress (MPa) | 14.91534 | 0.201654126 |

**Pre-buckling stress and strain values.** Maximum principal stress and strain energy values for all pre-buckling (linear static) simulations. Displacement and max. principal stress values are extracted from the highest values excluding the maximum contour around the load application area and averaged together.

**Table S3.**

| Model | Location | Parameter | Average | Standard Deviation |
|---|---|---|---|---|
| **Koch Iteration 1** | | | | |
| | Septal Plane | Total Strain (mJ) | 51.3628 | |
| | | Displacement (mm) | 0.8066495 | 0.000114441 |
| | | Max. Principal Stress (MPa) | 6575.373 | 125.3217054 |
| | Sutural Load | Total Strain (mJ) | 47.9447 | |
| | | Displacement (mm) | 0.8372084 | 0.000227 |
| | | Max. Principal Stress (MPa) | 11744.27 | 215.7194013 |
| **Koch Iteration 2** | | | | |
| | Septal Plane | Total Strain (mJ) | 103.507 | |
| | | Displacement (mm) | 1.011621 | 3.36006E-05 |
| | | Max. Principal Stress (MPa) | 12205.11 | 196.847268 |
| | Sutural Load | Total Strain (mJ) | 62.5973 | |
| | | Displacement (mm) | 0.7372091 | 2.37926E-05 |
| | | Max. Principal Stress (MPa) | 14741.45 | 268.8491185 |
| **Koch Iteration 3** | | | | |
| | Septal Plane | Total Strain (mJ) | 54.1105 | |
| | | Displacement (mm) | 0.6328125 | 2.14953E-05 |
| | | Max. Principal Stress (MPa) | 12892.93 | 253.3988952 |
| | Sutural Load | Total Strain (mJ) | 120.276 | |
| | | Displacement (mm) | 1.056672 | 2.89137E-05 |
| | | Max. Principal Stress (MPa) | 16919.84 | 341.3406457 |
| ***Spirula*** | | | | |
| | Inter-Chamber | Total Strain (mJ) | 271.25 | |
| | | Displacement (mm) | 0.4450942 | 0.000539138 |
| | | Max. Principal Stress (MPa) | 4627.16 | 26.1439603 |
| | Septal Attachment | Total Strain (mJ) | 133.619 | |
| | | Displacement (mm) | 0.1515158 | 9.35957E-05 |
| | | Max. Principal Stress (MPa) | 1987.228 | 46.51688764 |
| | Septal Attachment Base | Total Strain (mJ) | 90.6811 | |
| | | Displacement (mm) | 0.07903618 | 0.000196032 |
| | | Max. Principal Stress (MPa) | 725.068 | 36.1252909 |
| | Septal Plane | Total Strain (mJ) | 137.366 | |
| | | Displacement (mm) | 0.2722984 | 0.000214084 |
| | | Max. Principal Stress (MPa) | 2643.409 | 13.7037188 |
| ***Baculites*** | | | | |
| | Lobe | Total Strain (mJ) | 336.564 | |
| | | Displacement (mm) | 0.3169244 | 0.000291437 |
| | | Max. Principal Stress (MPa) | 3279.481 | 47.99450186 |
| | Septal Attachment | Total Strain (mJ) | 416.039 | |
| | | Displacement (mm) | 0.2261387 | 0.000397101 |
| | | Max. Principal Stress (MPa) | 2105.222 | 18.18432006 |
| | Mid Saddle | Total Strain (mJ) | 206.533 | |
| | | Displacement (mm) | 0.1721309 | 0.000923427 |
| | | Max. Principal Stress (MPa) | 2610.241 | 67.84742301 |
| | Saddle Tip | Total Strain (mJ) | 422.193 | |
| | | Displacement (mm) | 0.4246132 | 0.001549154 |
| | | Max. Principal Stress (MPa) | 5870.555 | 82.18508238 |

**Post-buckling stress and strain values.** Maximum principal stress and strain energy values for all post-buckling (non-linear general Riks) simulations. Displacement and max. principal stress values are extracted from the highest values excluding the maximum contour around the load application area and averaged together.

**Table S4.**

| ***Koch Models*** | *First Eigenvalue* | *Second Eigenvalue* | *Third Eigenvalue* |
|---|---|---|---|
| 2nd Iteration with roll constraints | 6.3680 | 7.0048 | 9.2400 |
| 2nd Iteration without roll constraints | 5.7444 | 6.3553 | 9.1187 |
